# Non-compliance and associated factors against smoke-free legislation among health care staffs in governmental hospitals in Addis Ababa, Ethiopia: an observational cross-sectional study

**DOI:** 10.1186/s12889-019-6407-z

**Published:** 2019-01-19

**Authors:** Tamiru Tadesse, Belay Zawdie

**Affiliations:** 1 Health Inspectorate Directorate, Ministry of Health, Addis Ababa, Ethiopia; 2 Department of Biomedical Sciences, Faculty of Medicine, Jimma University, Southwest, Jimma, Ethiopia

**Keywords:** Smoke-free, Hospital staff, Non-compliance, Legislation, Ethiopia

## Abstract

**Background:**

In 2014, the Ethiopian government passed a new smoking legislation that banned smoking in public and workplaces including health care facilities. However, data’s on level of non-compliance and associated factors with non-compliance towards smoke-free legislation in hospital settings of the country has not been studied yet.

**Methods:**

Hospital-based Cross-sectional study design triangulated with observational study was conducted in five hospitals. Data were collected through direct observation and interviews using checklist, structured and pre-tested questionnaires for observational study and survey of hospital employee respectively. Nine data collectors and one supervisor were involved in data collection. Three hundred fifty (350) health care staffs were interviewed. Fifteen (15) buildings were purposively observed for observational non-compliance in the selected hospitals. Data were entered by Epi Info and analyzed using SPSS version 21 software. Logistic regression was used to compute the crude and adjusted odds ratios for the factors affecting employee non-compliance with the legislation. A *p*-value of < 0.05 at 95% CI was considered to be statistically significant.

**Results:**

Anti-smoking signs were absent from a high proportion of hospital areas (97% overall) although visible cigarette butts were generally not observed in most areas of the hospitals. Non-compliance level among health care staffs was 50(10.3%).Associated factors affecting to the non-compliance level of the staff were: being male (AOR = 5.89, *p* value = 0.001), having poor knowledge (AOR = 2.71, *p*-value = 0.022) and having Unfavorable attitudes (AOR = 6.15, *p*-value = 0.000).

**Conclusions:**

Non-compliance level was high and needs careful implementation for 100% smoke-free legislation in addressing knowledge and attitudes of health care staffs.

## Background

Tobacco consumption and exposure to second-hand smoke (SHS) are a leading cause of morbidity and mortality worldwide. Cigarette smoking is the main source for SHS exposure. Second-hand tobacco smoke is the combination of smoke emitted from the burning end of a cigarette or other tobacco products and smoke exhaled by the smoker. Second-hand smoke contains over 7000 chemicals of which at least 69 are known carcinogens or otherwise toxic [1]. Moreover, it is firmly established that there is no safe level of exposure to tobacco smoke [2].

Globally, more than 7 million people die each year from tobacco-related illnesses (more than 6 million from direct tobacco use and approximately 890,000 non-smokers being exposed to secondhand smoke) [3], and if a current trends continue, this number is expected to increase to more than 8 million a year by 2030 [3]. In 2015, 6.4 million deaths were attributable to smoking worldwide [4]. Nearly80% of the world’s one billion smokers live in low- and middle-income countries. Among adults who worked indoors and/or outdoors in Ethiopia, 29.3% (6.5 million) were exposed to secondhand smoke in their workplace (Non-smokers, 27.1% or 5.7 million adults) while among adults who visited public places, 7.0% were exposed to the second-hand smoke in health-care facilities in which hospitals were one of the category [5].

As part of efforts to prevent this growing problem related to tobacco smoking, which can protect all peoples from the harmful effect of exposure to second-hand tobacco smoke, the World Health Organization’s Framework Convention on Tobacco Control (WHO FCTC) has called a comprehensive legislation to eliminate tobacco smoking in all indoor public places and workplaces and adapted the framework convention on Tobacco Control (FCTC) in 2003 [6, 7].

Following this, many countries in the WHO Africa Region are enacting and implementing domestic legislation for tobacco control [8]. Ethiopia is an early signatory in 2004 to the WHO FCTC, having ratified proclamation on February 17th, 2014 and entered into force on 23 June 2014 and adopted the tobacco control directive No. 28/2015; which comprehensively banned smoking in all public places including health care facilities which will add an input to the national tobacco control efforts and will assist our country in meeting its obligation under FCTC. This legislation specify that smoke-free public sites must post clear signs prohibiting smoking, removing ashtrays, ensuring compliance, requesting smokers to stop smoking or to leave the premises and notifying law enforcement agents. Despite such clear messages, the effectiveness of smoking-related policy and intervention may inevitably depend on the knowledge, attitudes, practice and support of health care staff to this legislation. However, the main challenge to the implementation of this legislation is that they are inadequately enforced or lately entered into enforcement [9, 10].

Health care facilities are among the most influential setting for modeling abstinence from smoking and encouraging smoke-free environment. Health professionals have the trust of the population, the media and opinion leaders, and their voices are heard across a vast range of social, economic and political arenas. Hospitals should promote, implement and comply with tobacco control legislation, particularly smoke-free legislation. However, this may not be an issue in Ethiopia, where the legislation smoke-free workplace enacted after the FCTC has yet to be enforced broadly across the region of the country and within health care facilities particularly in the hospitals settings [11].

In February17, 2014, the Ethiopian government passed a new smoking legislation through a house of parliament that banned smoking in public places and workplaces including health care facilities [12]. However, data’s on level of non-compliance and associated factors with smoke-free legislation in the hospital setting are unknown, since there is no study undertaken in Ethiopia yet. So, the aim of this study was to evaluate a level of non-compliance and identify its main predictor factors towards smoke-free legislation among health care staff in those selected hospitals.

## Methods

### Study areas and duration

This assessment was conducted in five hospitals located in the city government of Addis Ababa from June 1 to December 30, 2017. These hospitals include Tikur Anbessa general specialized hospital, St. Paul’s hospital millennium medical college, Amanuel mental specialized hospital, Alert hospital, and St. Peter specialized hospital. We choose these hospitals because they have started implementation of the smoke-free legislation in their hospital premises.

### Study design

A hospital-based Cross-sectional study design triangulated with the observational study was employed in five selected hospitals located in the city government of Addis Ababa, Ethiopia.

### Sample size determination

#### Hospital sample

Five hospitals located in the city government of Addis Ababa which have started the implementation of smoke-free legislation in their hospital premises were purposively included for this study. For the observational study data were collected by observing the 15 buildings located in the hospitals premises of five purposively selected hospitals according to the compliance guide [13].

#### Hospital health care staff sampling and procedure for interview

Three hundred and fifty four(354) employees were engaged from five selected hospitals by assuming confidence interval of 95%, a power of 80%,OR of 4.6 and ratio of 1:1 [14]. Employees were selected with the probability proportional to the size of different employee groups within the five hospitals by simple random sampling technique using random digit table. (Table 1).

**Table 1:** Proportional health care staffs selected for interview from each hospitals

Sr No	Hospital Name	Source population	Proportion (%)	Study population
1	Tikur Anbessa	2904	34	120
2	SPHMMC	2506	30	106
3	Alert	1307	15.4	55
4	Amanuel	1069	12.6	45
5	St. Peter	694	8	28
	Total	8480	100	354

### Appendix

**Second-hand smoke (SHS) exposure:** is secondary exposure of individuals to tobacco smoke as a result of others smoking tobacco.

**Smoke-free legislation**: is the legislation or policies/law which makes all or almost all workplaces, including hospital settings, totally smoke-free; with no smoking at all allowed in any indoor area.

**Observational non-compliance to the legislation:** Observation of at least one of the following anywhere in the hospital premises including; the presence of smoking, presence of cigarette butts, presence of ashtray and absence of anti-smoking sign.

**Employee non-compliance to the legislation**: is a cigarette smoking in the hospital premises by the employee.

**Compliance**: the degree to which a smoke-free legislation is being obeyed in the hospital premises.

C**urrent smoker:** is someone who had smoked cigarettes at least 6 months during their lifetime and was smoking tobacco products at the time of the survey [15].

**Former smoker:** is someone who had smoked at least 6 months during their lifetime but no longer currently smoke [15].

**Never smokers:** is someone who had never smoked cigarettes during their lifetime [15].

**Good Knowledge of smoke-free legislation:** health care staffs who have scored greater or equal to the groups mean score on the knowledge questions.

**Poor knowledge of smoke-free legislation**: health care staffs who have scored less than the groups mean score on the knowledge questions.

**Favorable attitude to smoke-free legislation**: health care staffs who have scored greater or equal to the groups mean score on the attitude questions.

**Unfavorable attitude to smoke-free legislation**: health care staffs who have scored less than the groups mean score on the attitude questions.

**Health care staffs:** all staffs working in hospitals; Administration staffs, health professionals, health- related and support staffs.

**Second-hand tobacco smoke:** the smoke emitted from the burning end of a cigarette or from other tobacco products usually in combination with the smoke exhaled by the smoker.

### Data collection instruments and procedures

Data were collected by using a checklist and well-structured questionnaire. An observational checklist was adapted from compliance guide [13]. A well-structured close ended face to face interview questionnaire was adapted from another similar setup [14]. We also included pertinent questions from a practical guide of establishing smoke-free hospitals [16]. The questionnaire prepared by English was translated into Amharic and back to English by lingual experts for checking correctness. Finally, Amharic translated questionnaire was provided to the study participants by nine data collectors. Site visits was done to observe non-compliance of the hospitals towards the legislation in the hospital premises during the peak visiting/busy hours[10:00 AM-12:30 AM at the morning and 2:00 PM to 4:30 PM after noon] as per compliance guide [13].

### Measures

In the selected hospitals, observation was done to the specific areas in the buildings like entrance, reception area, and patient waiting areas, patient wards, physicians’ room, nurses’ room, outpatient department, elevators, corridors, toilets, and café and administration offices. Indicators like presence of active smoking, presence of cigarette butt, and the presence of ashtray and absence of anti-smoking sign were counted, and then overall observational non-compliance level was estimated by dividing the number of areas that are non-compliant to the total number of eligible areas in each hospital.

Health care staff in those selected hospitals was asked about their non-compliance status [1 = yes, 0 = no] with the hospital smoke-free legislation during their working hours in the hospitals premises. Staff those self-identified as smokers was asked to indicate specific areas where they have been smoking during their working hours in the hospitals. Based on this employee non-compliance level was estimated by dividing the total number of employees who smoke in the hospital premises to the total number of surveyed employee.

To assess the knowledge of hospital health care staff, eight specific questions related to the smoke-free legislation were requested. Those questions were including, “exposure to tobacco smoke in the workplace is not a significant cause of tobacco-related diseases”, “designated smoking areas in the same room are effective in protecting non-smokers and workers from the hazards of tobacco smoke”, “separate rooms with separate ventilation offer almost the same amount of protection as smoking bans and are a good alternative to bans”, “tobacco product advertising has no effect on consumption”, “because of all the publicity about how harmful tobacco use is, anyone who starts using tobacco these days is fully aware of the risks”, “warnings/messages on tobacco product packages, while attention getting, are not effective in motivating users to quit”, “only smokers have to worry about health risks from tobacco smoke” and Smoke free legislation in hospital protects your health and/or the health of other visitors and patients. We created a scale using these items where 1 point was given for each correct answer with a possible score ranging from 0 to 8.A possible answer listed for each question was true or false [14] with the score greater or equal to the group mean score(> = 4.54) indicating having good knowledge and less than the group mean score indicated having poor knowledge towards the legislation [17].

To assess attitudes of hospital health care staff, a series of 11-questions related to smoke-free legislation was requested with five-point scale from “Strongly disagree to strongly agree”(0 = strongly disagree;1 = disagree 2 = unsure;3 = agree;4 = strongly agree) [14]. These questions were including, “a smoke-free hospital would improve the quality of care the patient receives”, “smoke from someone else’s cigarette is unhealthy for nonsmokers”, “the smoking habits of health professionals influence others”, “cessation programs should be offered to employees”, “hospital should be a smoke-free environment”, “hospital employees who work in offices or areas removedfrom direct patient care should be allowed to smoke”, “a smoke-free policy is hard to enforce”, “having a smoke-free policy is unfair to smokers”, “hospitals with smoke-free policies are likely to lose patients”, “smoking bans at hospitals would positively influence job performance” and “smoking bans at hospitals would positively affect the public image of the hospital”. A cumulative score was summed and yielded a possible range of 0–44 with score greater or equal to the group mean score(> = 29.78) indicating favorable attitude and less than the group mean score indicated unfavorable attitude towards the legislation [17, 18].

The practice of health care staff was assessed by asking one question about their practice in enforcing the legislation when a violation was observed in their hospital premises as per compliance guide [13]. Staffs those choose the statement “I/my manager would ask persons violating the legislation to stop smoking” and “I/my manager would call inspection authority” were considered as staffs who were practicing the enforcement of the legislation. Staffs those choose the statement “No action would be taken” and “I don’t know/I’m not sure” were considered as staffs those who were not practicing the enforcement of the legislation.

### Data quality management

Data collection tools were adapted from other literatures and prepared by a principal investigator. A well-structured questionnaire was translated into Amharic language and back-translated to the English language to maintain its consistency and corrective. Training was given for those data collectors and field supervisors on the objective of the study, contents of the questionnaire and how to maintain confidentiality and privacy of the participants. Finally, pre-test was performed at 5% of the sample size in AaBET hospital before the actual data collection. Data collection tools were revised, edited and modified according to the results of the pretest. A principal investigator and field supervisor had checked collected data for their completeness on the daily basis before the data were entered into the software. More orientation/guidance for those data collectors come up with incompleteness and inconsistency was provided in between data collecting period. In the case of the incompleteness and inconsistency, the collected data were discarded as incomplete.

### Statistical analyses

Observational data were analyzed by Microsoft Excel and univariate analysis was performed to estimate observational non-compliance level. Interviewed data were cleaned and entered into EPI info7 version software and analyzed by SPSSwindow version 21 and univariate analysis was also conducted to estimate employee non-compliance level in the selected hospitals. Bivariate and Multivariable analysis was applied using binary logistic regression to identify predicting factors with employee non-compliance towards smoke-free legislation. Variables at *P*-value less than 0.05 at 95% CI were considered as significant factors for non-compliance towards smoke-free legislation among hospital employees.

## Results

### Observational non-compliance with the smoke-free legislation

Almost at all inspected places (entrances, reception, waiting areas, patient wards outpatient clinics, corridors, elevators, stairs, physicians’ nurses’ and employees rooms) in five selected hospitals presences of “anti-smoking sign” was nonexistent. Only 2.8% of the inspected places have “anti-smoking sign”. The places where such signs were most likely absent were the physicians’ room, Nurses’ room, OPD, Elevators, and toilets. However, the place where this sign was more existent was at entrances (23.8%) compared to other places (Table 2).

**Table 2:** Distribution of anti-smoking signs and cigarette butts in five hospitals, 2017

Items inspected	Observed items in number				Total	Non-compliance
	Yes		No			
	No.	%	No.	%		
Presence of anti-smoking sign at;	16	2.8	555	97.2	571	97.2%
Entrance	5	23.8	16	76.2	21	
Patient waiting area	1	1.9	51	98.1	52	
Reception	1	12.5	7	87.5	8	
Patient wards	2	2.4	79	97.6	81	
Physicians’ room	0	0	48	100	48	
Nurses’ room	0	0	56	100	56	
OPD	0	0	87	100	87	
Corridors	1	1.5	63	98.5	64	
Elevators	0	0	2	100	2	
Toilets	0	0	61	100	61	
Café	3	20	12	80	15	
Administration offices	3	3.9	73	96.1	76	
Presence of cigarette butts at;	21	3.7	550	96.3	571	3.7%
Entrance	5	23.8	16	76.2	21	
Patient waiting area	2	3.8	50	96.2	52	
Reception	0	0	8	100	8	
Patient wards	0	0	81	100	81	
Physicians’ room	0	0	48	100	48	
Nurses’ room	0	0	56	100	56	
OPD	0	0	87	100	87	
Corridors	2	3.1	62	96.9	64	
Elevators	0	0	2	100	2	
Toilets	9	14.8	52	85.2	61	
Café	1	6.7	14	93.3	15	
Administration offices	2	2.6	74	97.4	76	
Other indicators	Yes		No		Total	
	No.	%	No.	%	No.(%)	
Presence of active smoking	3	60	2	40	5 (100)	60%
Presence of ashtray	0	0	5	100	5 (100)	0%
Presence of lighter/match box	0	0	5	100	5 (100)	0%
Presence of designated smoking areas	0	0	5	100	5 (100)	0%

Cigarette butts were more prevalent at the entrances (23.8%) and toilets (14.8%) compared to other places. Site visits indicated the evidence of smoking on the hospital premises was observed in 3 (60%) of the selected hospitals. However, cigarette butts were observed in all selected hospitals, which indicate evidence of recent cigarette smoking in these hospitals (Table 2).

### Socio-demographic characteristics of the respondents

A sample of 354 health care staffs was drawn from a population of 8480 health care staffs working in five hospitals located in the city government of Addis Ababa. Of these health care staffs, 4 were excluded due to incomplete filling of questionnaires. Responses were received from 350 participants indicating a response rate of 98.8%. Respondents were classified as current, former and never smokers. Accordingly, 14.3% of the samples were current smokers, 3.4% were former smokers and 82.3% were never smokers (Table 3).

**Table 3:** Socio, economic and demographic characteristic of the respondents per each hospitals, 2017

Demographic detail		Total Frequency		Tikur Anbessa (No.)	SPHMMC (No.)	Alert (No.)	Amanuel (No.)	St.peter (No.)
		No	%					
Sex	Male	190	54.3	74	53	21	25	17
	Female	160	45.7	43	52	34	20	11
Age	< 30 yrs	216	61.7	66	61	41	30	18
	30-39 yrs	82	23.4	27	34	6	9	6
	40-49 yrs	36	10.3	17	8	3	5	3
	50-59 yrs	13	3.7	5	2	4	1	1
	≥60 yrs	3	0.9	2	0	1	0	0
Marital status	Single	171	48.9	60	44	29	26	12
	Married	173	49.4	53	61	24	19	16
	Divorced/separate	4	1.1	3	0	1	0	0
	Widow	2	0.6	1	0	1	0	0
Profession	Health profession	200	57.1	83	46	25	28	18
	Health-related	9	2.6	5	1	1	2	0
	Admin	39	11.1	12	17	6	1	3
	Support	102	29.1	17	41	23	14	7
Job title	Specialist/clinical	166	47.4	55	42	25	28	16
	Researcher	3	0.9	0	3	0	0	0
	Admin	42	12	10	19	6	3	4
	Teaching	30	8.6	29	0	1	0	0
	General services	109	31.1	23	41	23	14	8
Type of employment	Contract	21	6	8	8	1	4	0
	Permanent	329	94	109	97	54	41	28
Smoking status	Never smoker	288	82.3	89	93	47	38	21
	Current smoker	50	14.3	20	12	8	4	6
	Former smoker	12	3.4	8	0	0	3	1
Working hours/week	≤40 h	182	52	77	6	43	34	22
	> 40 h	168	48	40	99	12	11	6
Monthly income	< 4708 birr	196	56	48	69	40	24	15
	> = 4708 birr	154	44	69	36	15	21	13

The median age of the respondents was 29 years (IQR 26 to 35 years) with the majority of the respondents (*n* = 216 or 61.7%) at a category of less than 30 years old. Of the 350 participants, 190(54.3%) were males. The majority of the study participants, 117 (30%), were from Tikur Anbessa General Specialized hospital. About 196(56%) of the respondent had a monthly income of less than 4708 birrs (mean monthly income). When considering the professional category of the study participants; the majority, (200; 57.1%) were health professionals, (9; 2.6%) were health-related staffs, (39; 11.1%) were administration staffs and (102; 29.1%) were support staffs. Among 350 respondents the majority, (*n* = 173; 49.4%) and (*n* = 171; 48.9%) of them were married and single respectively. One hundred eighty-two(52%) of the respondents worked less than or equal to 40 h per week (Table 3).

### Employee non-compliance level

**Fig. 1:**
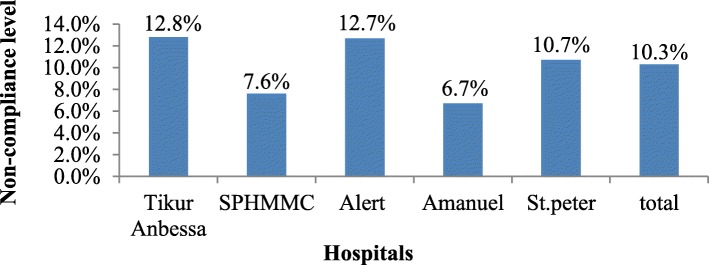
Distribution of employee non-compliance level per hospitals, 2017

Interview with hospital employee showed that there was a total of 10.3% non-compliance level observed among hospital employee in five selected hospitals. The highest level of non-compliance towards the legislation was observed among Tikur Anbessa hospital employee while the least was recorded in SPHMMC and Amanuel hospital employee (Fig. 1).

### Non-compliance status and attitudes of health care staff

Among compliant employee, 182(58%) and 168(53.5%) of them have a respectively good knowledge and favorable attitude towards smoke-free legislation and exposure to SHS. However, the majority of non-compliant employee, 25(69.4%) and 30(83.3%) have a respectively poor knowledge and unfavorable attitude towards smoke-free legislation and exposure to SHS (Table 4).

**Table 4:** Non-compliance status of health care staffs by knowledge and attitude in five hospitals (*n* = 350)

Non-compliance Status	Knowledge status		Total	Attitude status		Total
	Good	Poor		Favourable	Unfavourable	
Compliant	182 (58)	132 (42)	314 (100)	168 (53.5)	146 (46.5)	314 (100)
Noncompliant	11 (30.6)	25 (69.4)	36 (100)	6 (16.7)	30 (83.3)	36 (100)
Total	193 (55.1)	157 (44.9)	350 (100)	174 (49.7)	176 (50.3)	350 (100)

### Awareness of health care staff on the existence of smoke-free legislation

In all, 146(41.7%) of study participants were aware of the hospital smoke free legislation, 119(34%) were not aware and 85(24.3%) were not sure of the existence of the smoke-free legislation that prohibits smoking in the hospital premises. Among those aware of the hospital smoke-free legislation,14(9.6%) of the participants were non-compliant to the legislation. However, among non-compliant employees, 13(36.1%) were not aware of the legislation and 9(25%) of them were not sure of the existence of the legislation in the hospitals (Table 5).

**Table 5:** Awareness of health care staff on the existence of smoke-free legislation in five hospitals, 2017

Non-compliance status	Awareness to the existence of the smoke-free legislation			Total No. (%)
	Aware No. (%)	Unaware No. (%)	Unsure No. (%)	
Compliant	132 (90.4)	106 (89.1)	76 (89.4)	314 (89.7)
Noncompliant	14 (9.6)	13 (10.9)	9 (10.6)	36 (10.3)
Total	146 (41.7)	119 (34)	85 (24.3)	350 (100)

### Practice of the health care staff in enforcing smoke-free legislation

Only 98(28%) of the participants had been enforcing the smoke-free legislation. While the majority, 252 (72%), of them were not practicing the enforcement of the legislation (Table 6). According to their smoking profile; among smokers (*n* = 50), only 10% of them were practicing the enforcement of the smoke-free legislation and 45 (90%) them were not practicing when a violation was observed. Even the majority of non-smokers, 69%, were also not practicing the enforcement of the legislation (data not shown).

**Table 6:** Practice of health care staff by their non-compliance status in five hospitals, 2017

Non-compliance status	Enforcement status		Total
	Practice enforcement (%)	Not practicing Enforcement (%)	
Compliant	93 (94.9)	221 (87.7)	314 (89.7)
Non-compliant	5 (5.1)	31 (12.3)	36 (10.3)
Total	98 (28)	252 (72)	350 (100)

### Factors associated with employee non-compliance and multivariate analysis

Socio-demographic, Knowledge, attitude and practice of the study participants in relation to non-compliance were analyzed by bivariate analysis using simple binary logistic regression model. Binary logistic regression analysis was carried out to assess the relative effects of independent variables on the dependent variable. Those variables with *P*-value less than or equal to 0.20 were entered to the model. Next multivariable logistic regression was performed to assess associated risk factors to the non-compliance level of employee within hospital settings and this logistic regression analysis depicted that being male (AOR: 5.89, *p*-value:0.001), having poor knowledge (AOR:2.71,*p*-value:0.022) and having unfavorable attitude (AOR:6.15,*p*-value:0.000) were the three significant risk factors associated with non-compliance level of health care staffs towards the smoke-free legislation within the selected five Hospitals (Table 7).

**Table 7:** Bivariate and Multivariable analysis for risk factors associated with non-compliance of the employee in five hospitals, 2017

Risk factors	Category	Non-compliance status		Bivariate analysis			Multivariable analysis		
		Yes	No	COR	95% CI	*p*-value^*^	AOR	95% CI	*p*-value^*^
Sex	Female	6	154	1(Ref.)			1(Ref.)		
	Male	30	160	4.81	1.95–11.88	0.001	5.89	2.08–16.64	0.001
Age	< 30 yrs	17	199	1(Ref.)			1(Ref.)		
	30-39 yrs	7	75	1.09	0.44–2.74	0.85	0.44	0.14–1.41	0.167
	40-49 yrs	10	26	4.50	1.87–10.87	0.001	3.04	0.83–11.20	0.094
	50-59 yrs	2	11	2.13	0.44–10.39	0.350	2.96	0.46–19.07	0.253
	> = 60 yrs	0	3	0	0.00	0.999	0.00	0.00	0.999
Marital status	Single	13	158	1(Ref.)			1(Ref.)		
	Married	22	151	1.77	0.86–3.64	0.120	1.45	0.51–4.11	0.488
	Separate/divorce	1	3	4.05	0.35–41.75	0.24	0.62	0.04–9.96	0.732
	Widow	0	2	0	0.00	0.99	0.00	0.00	0.999
Type of employment	Contract	5	16	1(Ref.)			1(Ref.)		
	Permanent	31	298	0.33	0.11–0.97	0.044	0.39	0.10–1.48	0.167
Practice	Enforcing	5	93	0.38	0.15–1.02	0.054	0.39	0.13–1.16	0.089
	Not enforcing	31	221	1(Ref.)			1(Ref.)		
Attitude status	Favorable attitude	6	168	1(Ref.)			1(Ref.)		
	Unfavorable attitude	30	146	5.75	2.33–14.21	0.000	6.15	2.26–16.69	0.000
Knowledge status	Good knowledge	11	182	1(Ref.)			1(Ref.)		
	Poor knowledge	25	132	3.13	1.49–6.59	0.003	2.71	1.15–6.36	0.022

^*^Binary logistic regression

## Discussion

The purpose of this study was to estimate observational non-compliance level, employee non-compliance level and associated factors towards the smoke-free legislation in hospital settings among hospital health care staff, by assessing the knowledge, attitudes, practice, and smoking habits of health care staff towards the smoke-free legislation. Observational non-compliance was also assessed by observing the hospital premises towards the legislation. This study showed non-compliance level among health care staffs was high (Fig. 1) and affected by their sex, knowledge, and attitudes towards the legislation. Site visits also indicated that observational non-compliance level towards the legislation was high (Table 2).Thus, the non-compliance level shown here may help the enforcement authority to strictly see the implementation of the legislation in health care facilities of Ethiopia.

Non-compliance level among health care staffs shown here was higher than non-compliance levels reported in the UK [19] and lower than non-compliance levels reported in Portugal [20] and teaching hospital in Spain [21]. The higher proportion may be due to unfavorable attitudes of health care staffs towards the smoke-free legislation, because this study revealed above half of the health care staffs had unfavorable attitude; even the majority of smokers had also unfavorable attitude towards the smoke-free legislation. The other reason may be because the implementation of smoke-free legislation was lately started in the hospital setting of our country than in the UK. This result indicated non-compliance level of health care staffs can be minimized by creating awareness on the legislation using different mechanisms like training.

Site visits also indicated observational non-compliance level was high in the premises of the hospitals as per WHO FCTC guideline and indicator of success [22, 23]. This was affirmed by the presence of cigarette butts, active smoking, and absence of anti-smoking sign in all appropriate places of the hospitals. These findings was not in line with findings reported in acute care hospitals of US [24] in which non-compliance of surveyed hospitals with the JCAHO national smoke-free policy was low compared to the current findings. This site visits further implies observational non-compliance level was high compared to the individual non-compliance level. The high level of observational non-compliance shown here was not consistent with different research findings reported in different countries [9, 14, 18]. The reason for the high proportion of observational non-compliance in the hospital premises may be due to the fact that Ethiopia has adapted WHO FCTC and lately entered into enforcement than the above two counties [12]. However, the reason for a low proportion of the existence of cigarette butts in the current study is may be due to the low proportion of cigarette smoking staff than the above three studies [9, 14, 18]. Other reason may be attributable to the janitorial staff cleaning hospital areas during the morning time which couldn’t be observed while data collection. Even-though hospitals observed with the presence of persons smoking a cigarette were not in all five selected hospitals, there was a cigarette butt observed in all five selected hospitals which further indicates there was an evidence of recent cigarette smoking in all observed hospitals. The proportion of hospitals that actually observed with active smoking was higher in the current study than the proportion reported in the Turkey [18]. This may be due to the problems related to the staff attitude and awareness towards the existence of smoke-free legislation in the hospitals.

Logistic regression analysis indicated sex, knowledge, and attitudes of health care staff were the three positively associated factors with non-compliance level of health care staff towards smoke-free legislation. Being a male is almost six times more likely to be non-compliant than being a female, having a unfavorable attitudes six times more likely to be non-compliant than having a favorable attitude while having poor knowledge is almost three times more likely to be non-compliant than having good knowledge towards the legislation.

The significant association of health care staffs’ sex with their non-compliance level in the current study was not consistent with research done in the United Kingdom [11]; in which sex of hospital employee was not significantly associated with smoke-free policy. In our current study, it may be because the prevalence of cigarette smoking was high among male than female which is also high among male than female in the STEPS survey of 2015 [25] and EDHS 2011 [26].

Different studies [27–31] pointed that non-compliance of individuals with smoking ban was affected by their knowledge which is in line with our current study findings that health care staffs’ knowledge has a positive association with their non-compliance towards smoke-free legislation. Peoples who were better informed with the harms of smoking and passive smoking and aware of policy were more likely to be compliant with the smoking bans in public places than those with a lower level of knowledge. This association maybe due to the fact that implementation of the legislation was lately started and lack of awareness creation on the smoke-free legislation. Additionally, other studies [27, 29, 30] conducted in different countries on smoke-free policy was in line with our current findings. In these studies, knowledge of the surveyed staffs was positively associated with the smoke-free policy. Similarly, it was also consistency with research done in Australia in 2010 [31] and in China in 2010 and 2014 [28, 32] in which knowledge of the surveyed staffs has also a positive association with the smoke-free policy.

Regarding the significance of hospitals health care staffs’ attitude with their non-compliance level, different studies [27, 33] pointed that unfavorable attitude towards smoking bans and exposure to SHS was also identified as a key factor affecting to non-compliance of individuals with the smoking bans. People who had unfavourable attitudes towards smoking bans in public places and exposure to SHS were less likely to be compliant with the smoke-free legislation, which is in line with our current findings. Thus, the findings shown here indicated a more effort is needed by enforcement authority and hospital administrators in implementing smoke-free legislation.

Although this study has important findings, it has some limitations related to its cross-sectional design like recall bias and the reliance on self-reported data. Especially, non-compliance status of hospital employee was based on what they self-reported during working hours which may make over or under report. We had also focused on the federal government hospitals due to budget limitations; which might not reflect non-compliance level in other health care facilities of the country. Being the first study in the hospital setting of our country is also another limitation which we are unable to compare and contrast current findings with others.

## Conclusions

Findings of this study showed that non-compliance level among health care staff was high, which implies the legislation has not been fully implemented as per WHO FCTC guideline. Especially, observational non-compliance level towards the legislation was high compared to the individual non-compliance level. The factors associated with the non-compliance level of health care staffs were; being male, having poor knowledge and unfavorable attitudes towards the smoke-free legislation. This study suggests careful implementation of the legislation was needed in addressing the knowledge and attitudes of health care staffs.

## Abbreviations

AaBET: Addis Ababa Burn, Emergency and Trauma
AOR: Adjusted odds ratio
CI: Confidence interval
COR: Crude odd ratio
EDHS: Ethiopian Demographic Health Survey
IQR: Inter quartile range
IRB: Institutional review board
OPD: Out patient department
OR: Odds ratio
SHS: Second hand smoke
SPHMMC: St. Paulo’s Hospital Millennium Medical College
SPSS: Statistical Package for Social Science
STEPs: The STEPwise approach to non-communicable disease risk factors surveillance
UK: United Kingdom
US: United State
WHO: World Health Organization
WHOFCTC: World Health organization Framework Convention on Tobacco Control
